# Cross-Border Investigations on the Prevalence and Transmission Dynamics of *Cryptosporidium* Species in Dairy Cattle Farms in Western Mainland Europe

**DOI:** 10.3390/microorganisms9112394

**Published:** 2021-11-20

**Authors:** Pedro Pinto, Cláudia A. Ribeiro, Sumaiya Hoque, Ourida Hammouma, Hélène Leruste, Sébastien Détriché, Evi Canniere, Yvonne Daandels, Martine Dellevoet, Janine Roemen, Anne Barbier Bourgeois, Martin Kváč, Jérôme Follet, Anastasios D. Tsaousis

**Affiliations:** 1 Laboratory of Molecular and Evolutionary Parasitology, RAPID Group, School of Biosciences, University of Kent, Canterbury CT2 7NZ, UK; pp410@kent.ac.uk (P.P.); C.Azevedo-Ribeiro@kent.ac.uk (C.A.R.); sh986@kent.ac.uk (S.H.); 2 UMR-Transfrontalière 1158 BioEcoAgro, Junia, University of Lille, University of Liège, UPJV, ULCO, University of Artois, INRAE, F-59000 Lille, France; ourida.hammouma@junia.com; 3 Junia, Comportement Animal et Systèmes d’Elevage, F-59000 Lille, France; helene.leruste@junia.com; 4 University of Lille, Institut Mines-Télécom, University of Artois, Junia, ULR 4515—LGCgE, Laboratoire de Génie Civil et Géo-Environnement, F-59000 Lille, France; sebastien.detriche@junia.com; 5 Inagro vzw, Ieperseweg 87, 8800 Rumbeke-Beitem, Belgium; evi.canniere@inagro.be; 6 Southern Agricultural and Horticultural Organisation (ZLTO), Onderwijsboulevard 225, 5223 DE’s-Hertogenbosch, The Netherlands; yvonne.daandels@zlto.nl (Y.D.); martine.dellevoet@zlto.nl (M.D.); janine@agrisyst.com (J.R.); 7 Selas, 31 Rue de la République, 59496 Salome, France; anne.barbier@selascve.fr; 8 Biology Centre of the Academy of Sciences of the Czech Republic, Institute of Parasitology, 37005 České Budějovice, Czech Republic; kvac@paru.cas.cz; 9 Faculty of Agriculture, University of South Bohemia in České Budějovice, 37005 České Budějovice, Czech Republic; 10 University of Lille, CNRS, Centrale Lille, Junia, University Polytechnique Hauts de France, UMR 8520 IEMN Institut d’Electronique de Microélectronique et de Nanotechnologie, F 59000 Lille, France; jerome.follet@junia.com

**Keywords:** *18S* rRNA, *Cryptosporidium*, dairy cattle, *gp60*, genotyping, prevalence

## Abstract

*Cryptosporidium* is an apicomplexan parasitic protist, which infects a wide range of hosts, causing cryptosporidiosis disease. In farms, the incidence of this disease is high in animals such as cows, leading to extensive economic loss in the livestock industry. Infected cows may also act as a major reservoir of *Cryptosporidium* spp., in particular *C. parvum*, the most common cause of cryptosporidiosis in these animals. This poses a risk to the trading of livestock, to other farms via breeding centres, and to human health. This study is a part of a global project aimed at strategies to tackle cryptosporidiosis. To reach this target, it was essential to determine whether prevalence was dependent on the studied countries or if the issue was borderless. Indeed, *C. parvum* occurrence was assessed across dairy farms in certain regions of Belgium, France, and the Netherlands. At the same time, the animal-to-animal transmission of the circulating *C. parvum* subtypes was studied. To accomplish this, we analysed 1084 faecal samples, corresponding to 57 dairy farms from all three countries. To this end, *18S* rRNA and *gp60* genes fragments were amplified, followed by DNA sequencing, which was subsequently used for detection and subtyping *C. parvum*. Bioinformatic and phylogenetic methods were integrated to analyse and characterise the obtained DNA sequences. Our results show 25.7%, 24.9% and 20.8% prevalence of *Cryptosporidium* spp. in Belgium, France, and the Netherlands respectively. Overall, 93% of the farms were *Cryptosporidium* positive. The *gp60* subtyping demonstrated a significant number of the *C. parvum* positives belonged to the IIa allelic family, which has been also identified in humans. Therefore, this study highlights how prevalent *C. parvum* is in dairy farms and further suggests cattle as a possible carrier of zoonotic *C. parvum* subtypes, which could pose a threat to human health.

## 1. Introduction

*Cryptosporidium* is a genus of enteric apicomplexan parasites, responsible for causing cryptosporidiosis in a diverse range of vertebrate hosts, including livestock and humans [1,2]. Cryptosporidiosis is one of the prominent causes of diarrheal illness in humans and animals worldwide, with children, newborn animals and immunocompromised individuals being especially vulnerable to the disease [3,4,5]. *Cryptosporidium* infections occur after ingestion of oocysts through the fecal–oral route, either directly after contact with animals infected or incidentally through contaminated material such as food, water, soil and fomites [6,7]. Thus, a One-Health approach, where all these factors are considered, is essential to investigate the role and transmission dynamics of this parasite in both humans and other animals [7].

In livestock, cryptosporidiosis primarily manifests as a gastrointestinal disease, causing watery diarrhea, malnutrition, abdominal pain, dehydration and, in severe cases, death [7,8]. This disease is deemed globally endemic in cattle and it is particularly prevalent in neonatal and pre-weaned calves (<6 weeks old), in which it is one of the most common causes of diarrheic illness [9,10]. *Cryptosporidium* infection and consequent disease appear in both beef and dairy calves, with higher prevalence in intensive livestock management systems [11]. Clinical manifestations of cryptosporidiosis in calves lead to long term impacts on their well-being and consequently, the disease puts a considerable economic burden on the cattle industry [9,10,12]. Costs associated with the economic burden of the disease include seeking veterinary expertise for diagnosis and medication, along with additional costs linked to animal rearing and supplemental nutrition to regain meat and milk yield. An additional cost of purchasing new animals has to be incurred in case of death [13]. In the United Kingdom, the extra cost to manage diarrheal diseases, including *Cryptosporidium*, has been estimated at an average of GBP 32 per calf with an annual total spending of GBP 11 million [13,14]. More recently, the economic burden of cryptosporidiosis in calves was highlighted, with projections reaching GBP 100–200 per *Cryptosporidium*-infected calf. The long-term difference in the growth of beef calves with and without cryptosporidiosis was also assessed [10,15]. An average variation of 34 kg was observed between the two groups, with infected calves being considerably lighter than the uninfected, translating into a profit deficit of approximately GBP 128 per animal according to market prices in 2018 [10]. Hence, studying the prevalence and transmission dynamics of this parasite in cattle farming is crucial. 

*Cryptosporidium* spp. incidence in cattle is mostly attributed to four species: *C. andersoni, C. bovis, C. parvum,* and *C. ryanae* [3]. Remarkably, an age-related scattering pattern has been noted, with *C. bovis* and *C. ryanae* being prevalent in post-weaned calves, while *C. andersoni* being the main infective species in adults [13,16,17,18,19,20,21,22]. The latter three species have non or low pathogenic potential and seem to be host-adapted [23,24,25]. In contrast, *C. parvum* is the main source of infection in pre-weaned calves and the only species associated with typical cryptosporidiosis symptoms in cattle [7,17,19,26,27]. Most notably, *C. parvum* is capable of infecting multiple animal hosts and is the primary zoonotic agent of cryptosporidiosis [5,28]. Hence, it is of particular concern that, during the infective stage, calves can shed an extraordinary number of oocysts in their feces (roughly 10 × 10^10^, per day) [29]. Since oocysts are extraordinarily resistant to a vast array of conditions, including common disinfectants, they can persist in the environment for long periods, and become readily infective after ingestion by humans and other animals [30,31,32]. 

To truly uncover the species diversity, zoonotic potential, and transmission dynamics of the different *Cryptosporidium* spp. circulating in cattle, molecular tools must be employed. By targeting and amplifying the *18S* ribosomal RNA gene followed by sequencing, it is possible to accurately distinguish and characterize *Cryptosporidium* at the species level [2,33]. Subsequently, amplification of the 60 kDa glycoprotein gene (*gp60*) can be used to explore the intra-specific diversity of *C. parvum* in order to categorize it into different subtype families and further differentiate subtypes within the same family [2,33,34]. These molecular tools have been extensively employed to assess *C. parvum’s* role in zoonotic transmission and to trace sources of cryptosporidiosis outbreaks [6,35]. *Cryptosporidium* spp. prevalence studies with further molecular characterization in cattle farms across Belgium, France and the Netherlands are quite sparse. Several reports have documented *Cryptosporidium* spp. occurrence and description of zoonotic species/subtypes in dairy or beef calves employing molecular tools in France [36,37,38,39,40,41,42]. To our knowledge, only one such study has been conducted in Belgium [43] and the Netherlands [44]. Therefore, the aim of the present study was to provide up-to-date information regarding the prevalence and transmission dynamics of *Cryptosporidium* species in cattle farms on a wide geographic area across Belgium, France, and the Netherlands, while subsequently investigating potential circulation of subtypes within and between farms and even countries.

## 2. Material and Methods

### 2.1. Geographical Area of Research and Study Model

This study was conducted under the Health for Dairy Cows (H4DC) project, funded by the Interreg 2 seas programme (https://h4dc-interreg2seas.eu/; accessed on 20 November 2021). This is a European territorial cooperation program covering the regions along the Southern North Sea and the Channel. This includes certain districts, such as the Flanders region of Belgium, the south of England, the Hautes-de-France region in France, and the west part of the Netherlands. The main objective of the project is to reduce the sanitary and economic impact of *Cryptosporidium* spp. on farms.

### 2.2. Faecal Sample Collection

**Figure 1:**
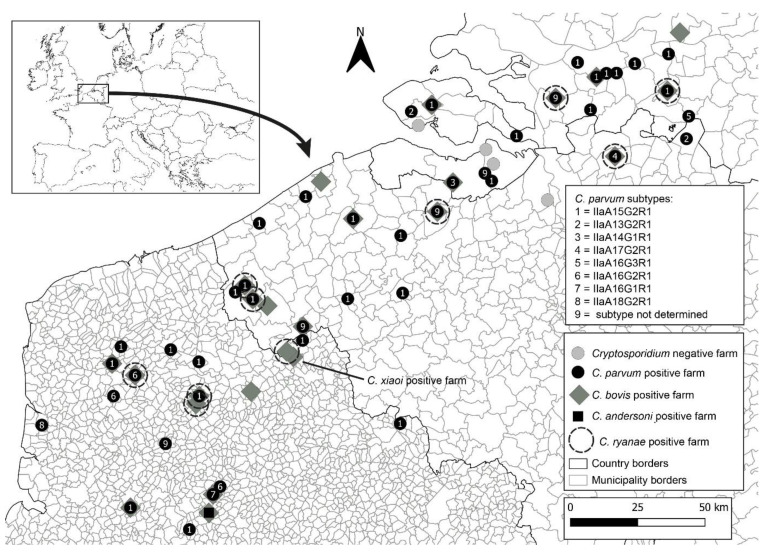
Geographical map of Belgium, France and the Netherlands indicating farms negative for *Cryptosporidium* spp. (grey circles) and farms positive for *C. parvum* (black circles), *C. bovis* (grey diamonds), and *C. andersoni* (black square). Distribution of *C. parvum* isolates with gp60 subtypes are represented as numbers.

Faecal sample collection for all participating farms in this study occurred between September 2019 and June 2020. Fifty-seven farms were surveyed from three countries: Belgium, France and the Netherlands (Figure 1). The partners of the project selected the participating farms. Most farmers participated in this study because of one of the following reasons: (1) the dairy cows in their farms were encountering issues with diarrhoea; (2) they were willing to participate in the study regardless; and/or (3) they were willing to potentially change their farming practices. On each farm, veterinarians were instructed to collect 10 individual faecal samples, preferentially from the youngest calves up to three months of age and 10 individual faecal samples from their respective mothers, directly from the rectum and regardless of their clinical condition (diarrhoeic or non-diarrhoeic). Thus, on average, a total of 20 samples were collected per farm. For farms with less than 10 calves ≤3 months old, fewer samples of calves (and their mothers) were collected. All participating farms were visited once. In one of the farms in Belgium, a total of 41 samples were collected (20 calves and 21 adults). Each sample was gathered using a single pair of disposable gloves, transferred to a 40 mL faecal collection container, and stored in a portable cooler until arriving to the storage place. Samples were stored at −20 °C until finally shipped on ice packs to the laboratories for further analysis. All samples contained the animal identification number, the relationship status between calf and mother, as well as, the date of sampling, age of the animal, farm identification and country of origin.

### 2.3. Sample Processing and DNA Extraction

Frozen faecal samples were thawed overnight at 4 °C and approximately 200 mg of faecal material was used to extract genomic DNA (gDNA) with the PureLink™ Microbiome DNA Purification Kit (Thermo Fisher Scientific, Carlsbad, CA, USA), according to the manufacturer’s commercial protocols with slight modifications. After the addition of clean-up buffer and instant vortex, samples were left to incubate at the fridge for 10 min to improve the removal of PCR inhibitors. DNA was then recovered with elution buffer and stored at −20 °C until further analysis.

### 2.4. Cryptosporidium spp. Detection, and Subtyping

*Cryptosporidium* spp. detection was attained through a nested PCR reaction amplification of the *18S* rRNA gene sequence. The primary reaction was conducted using the primers CRY_SSU_F1 5′-GATTAAGCCATGCATGTCTAA-3′ and CRY_SSU_R1 5′-TTCCATGCTGGAGTATTCAAG-3′ (product size: 723 bp) and the secondary reaction of the nested PCR was conducted using the forward primer CRY_SSU_F2 5′ -CAGTTATAGTTTACTTGATAATC- 3′ and the reverse primer CRY_SSU_R2 5′-CCTGCTTTAAGCACTCTAATTTTC- 3′ (product size ~631 bp) [45]. The primary PCR mixture was performed in a 25 μL volume containing 1 μL of template gDNA (concentration 10–100 ng/μL), 0.4 μM of each primer, 12.5 μL of 2 × PCRBIO Taq Mix Red (PCR Biosystems, London, UK). All amplifications were performed in a C1000 Touch PCR thermal cycler (Bio-Rad Laboratories, Inc., Berkeley, CA, USA) with an initial denaturation at 94 °C for 2 min, followed by 24 cycles of denaturation at 94 °C for 50 s, annealing at 53 °C for 50 s, and extension at 72 °C for 1 min. Lastly, a final extension step at 72 °C for 10 min was also included. For the second PCR reaction, 1 μL of the PCR product obtained from the first PCR reaction was used as a template, and the remaining of the mixture was prepared as described above for the primary reaction. The cycling conditions for the second PCR reaction differed on the number of cycles, with 30 cycles instead of 24, and the annealing conditions, with 56 °C for 30 s being used instead. Amplification of the *gp60* by nested PCR was carried out using the primers AL3531 5′ -ATAGTCTCCGCTGTATTC- 3′ and AL3535 5′ -GGAAGGAACGATGTATCT- 3′ (product size: 1000 bp) in the primary reaction of the nested PCR, and the primers AL3532 5′-TCCGCTGTATTCTCAGCC- 3′ and AL3534 5′ -GCAGAGGAACCAGCATC- 3′ (~850 bp) in the secondary reaction of the nested PCR [46]. Briefly, the primary and secondary PCR mixtures contained 2 μL of gDNA (ranging from 10 to 100 ng/μL) or of the primary PCR product, respectively, 0.2 μM of each primer, and 15 μL of 2 × PCRBIO Taq Mix Red (PCR Biosystems, United Kingdom) in a total volume of 30 μL. The cycling conditions included an initial denaturation step at 94 °C for 3 min, followed by 35 cycles of denaturation at 94 °C for 45 s, annealing at 50 °C for 45 s, and extension at 72 °C for 1 min. Lastly, a final extension step at 72 °C for 7 min was also included. Nuclease-free water and gDNA extracted from 10^6^ of *Cryptosporidium parvum* IOWA strain purified oocysts (Waterborne^TM^, Inc., New Orleans, LA, USA) were used as a negative and positive control, respectively.

Secondary PCR products were separated by electrophoresis in a 2% (*w*/*v*) agarose gel (2%) stained with ethidium bromide (0.2 μg/mL), and visualised under a UV light system (Syngene G:BOX Chemi XX6, UK). Bands of interest were excised from the gel and the DNA was purified using Thermo Scientific GeneJET Gel Extraction Kit (Thermo Fisher Scientific, CA, USA) following the manufacturer’s instructions.

### 2.5. Sequencing and Phylogenetic Analysis

Bi-directional sequencing of the purified secondary PCR products was outsourced to Eurofins (UK), who performed Sanger sequencing with the set of the primers used for the secondary PCR reaction. The quality of both forward and reverse nucleotide sequences generated for the expected amplicons were then manually assessed and trimmed if necessary, with ChromasPro version 2.1.9 (http://technelysium.com.au/wp/chromaspro/; accessed on 1 November 2020), assembled into a consensus sequence and mismatches corrected with the same software. The final assembled consensus sequence was then compared with GenBank reference sequences using the Basic Local Alignment Search Tool (BLAST) from the National Center for Biotechnology Information (NCBI) (http://blast.ncbi.nlm.nih.gov/Blast.cgi; accessed on 1 November 2020).

The sequences generated in this study were aligned with each other and with reference sequences from GenBank by MAFFT v.7 (https://mafft.cbrc.jp/alignment/server; accessed on 15 January 2021) and the sequence alignment was manually inspected with BioEdit version 7.2.5 (https://bioedit.software.informer.com; accessed on 15 January 2021). Phylogenetic analyses were performed and best DNA/Protein phylogeny models were selected using the MEGAX software [47,48]. Phylogenetic trees were inferred using maximum likelihood (ML), with the substitution model that best fit the alignment selected using the Bayesian information criterion. The Tamura 3-parameter model [49], was selected for *SSU* and *gp60* alignments. Bootstrap support for branching was based on 1,000 replications. Phylograms were drawn using MEGAX and were manually adjusted and annotated using CorelDrawX7.

*C. parvum* allelic family and subtype was identified from the partial sequence of *gp60* gene based on the subtypes nomenclature reported previously [34]. The *18S* rRNA and *gp60* sequences derived in this study were deposited in GenBank. The accession numbers are: MW947282-MW947436 for *18S* rRNA of *C. parvum*, MZ021416 for *C. xiaoi*, MZ021417-MZ021429, MZ021431, MZ021433-MZ021436, MZ021438-MZ021441, MZ021443, MZ021445, MZ021447, MZ021449, MZ021451- MZ021461, MZ021464-MZ021467 and MZ021469-MZ021470 for *18S* rRNA for *C. bovis*, MZ021426 for *C. andersoni* and MZ021430, MZ021432, MZ021437, MZ021442, MZ021444, MZ021446, MZ021448, MZ021450, MZ021462-MZ021463, MZ021468 and MZ021471 for *C. ryanae*. The accession numbers MW996760-MW996892 are for the *gp60* of *C. parvum*.

## 3. Results

### 3.1. Sampling Report

In this study, a total of 1084 faecal samples from 57 farms were collected. These included 545 faecal samples from dairy calves and 539 from cows. Twenty of these were located in the Netherlands, 20 in France, and 17 in Belgium (Figure 1). For each farm, between 6 and 41 faecal samples were collected (median 20 and mean of 18.4 ± 4.4) and a roughly even ratio of faecal sample collection from calves and their corresponding mother cows was obtained. The age of the tested calves varied from 0 to 106 days (14 median and mean of 21.6 ± 21.1)

### 3.2. Cryptosporidium spp. Occurrence and Prevalence in Farms across Belgium, France, and the Netherlands

Using amplification of a partial *18S* rRNA gene fragment with nested PCR revealed at least one animal tested positive for *Cryptosporidium* in 94.1% (*n* = 17) farms in Belgium, 100% (*n* = 20) in France and 85.0% (*n* = 17) in the Netherlands. Considering all farms, the overall occurrence of *Cryptosporidium* at the farm level was 93.0% (53/57).

Within Belgium, *Cryptosporidium* spp. infections ranged from 5.0% (1/20 cows, farm 9) to 40.0% (8/20 cows, farm 11). Infection rates ranged between 5.6% (1/18 cows, farm 18) to 50.0% (3/6 cows, farm 31) in France, while in the Netherlands rates varied between 5.0% (1/20 cows, farm 51) and 30.0% (6/20 cows, farms 46, 50, 56 and 57) (Table 1).

In terms of animals, 63 out of 335 were positive for *Cryptosporidium* spp. (18.8%) in Belgium, with young calves exhibiting a higher susceptibility (31.2%; *n* = 170) to infection as opposed to adults (6.1%; *n* = 165). In France, 79 out of the 350 (22.6%) animals tested positive with a prevalence of 41.1% (*n* = 175) in calves as opposed to 4.0% prevalence in adults (*n* = 175). Lastly, in the Netherlands’ farms, 69 out of 399 animals (17.3%) were positive, with 32.5% (*n* = 200 prevalence in calves compared to adults, 2.0% (*n* = 199) (Table 2).

#### 3.2.1. Belgium

**Figure 2:**
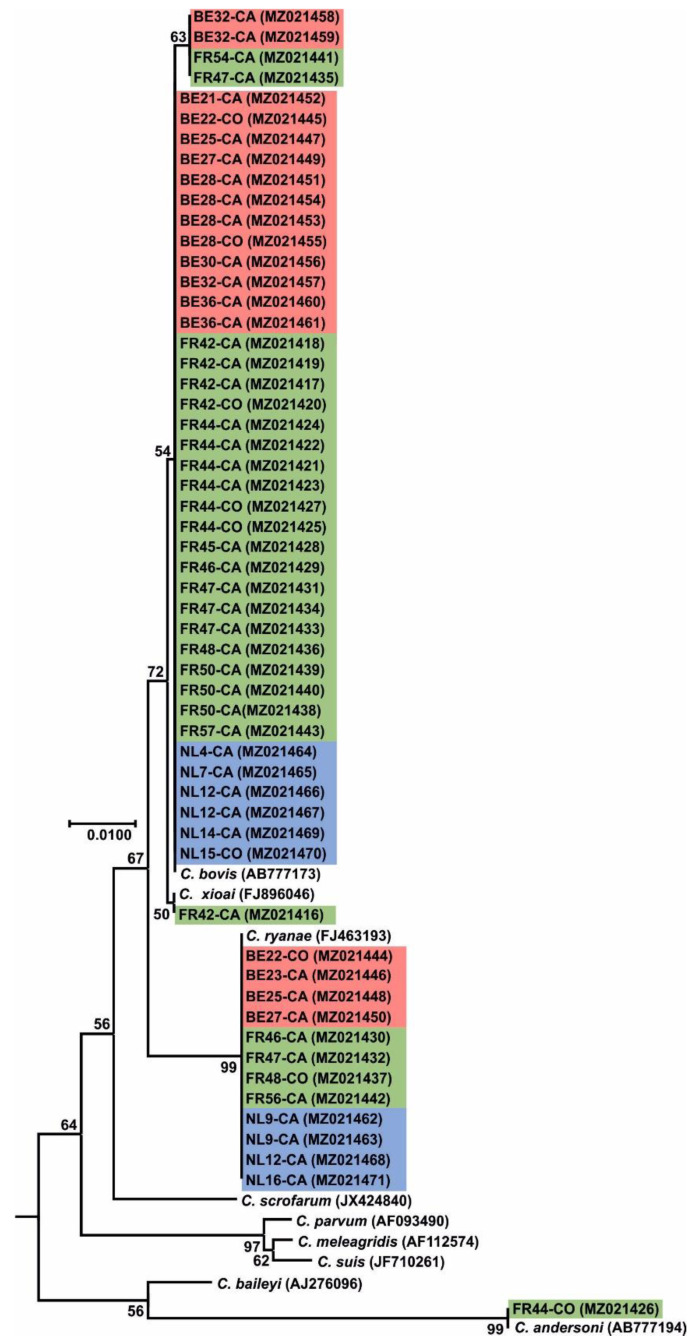
A maximum likelihood (ML) tree based on the 18S rRNA gene sequences of *C. bovis*, *C. ryanae*, *C. xioai* and *C. andersoni* obtained in this study. Bootstrap values for the nodes with more than 50% support are shown. Sequences from this study are identified by country (NL for the Netherlands and highlighted in blue; BE for Belgium and highlighted in red; FR for France and highlighted in green), number of the farm (e.g., NL4), and host age (CA for calf and CO for cow). The ML tree was rooted with an 18S rRNA sequence from Monocystis agilis (accession number: AF457127). The GenBank accession number for each sequence is mentioned in parenthesis.

In Belgian farms, 63 animals were identified as *Cryptosporidium* spp. with 45 cases (71.4%) identified as *C. parvum*, 14 (22.2%) as *C. bovis* and four as *C. ryanae* (6.4%). In calves *C. parvum* was identified in 38 cases (73.1%), while 12 (23.1%) were shown to be *C. bovis*, and three (5.8%) were assigned as *C. ryanae*. In adult dairy cows, we identified seven positives (70.0%) for *C. parvum*, two (20.0%) for *C. bovis* and one for *C. ryanae* (10.0%) (Table 2). Sequence analysis of the *18S* rRNA for *C. parvum* isolates revealed 100% nucleotide identity to the AH006572 reference sequence for 44 sequences (accession numbers MW947333-MW947340 and MW947342- MW947377) and 99% nucleotide identity to AH006572 reference sequence for the remaining sequences detected (accession number MW947341, displaying an A-to-T transversion at position 223, and an A-to-G transition at position 562). Sequence analysis of the *C. bovis* isolates, revealed 100% nucleotide identity to the AB777173 reference sequence for 12 sequences (MZ021445, MZ021447, MZ021449, MZ021451-MZ021457 and MZ021460-MZ021461) and 99% nucleotide identity to the AB777173 reference sequence for two sequences (MZ021458- MZ021459). *C. ryanae* isolates exhibited a 100% nucleotide identity to the reference sequence FJ463193 for four sequences (MZ021444, MZ021446, MZ021448 and MZ021450) (Figure 2).

#### 3.2.2. France

In France, a total of 79 samples tested positive for *Cryptosporidium* spp. with *C. parvum* being identified in 51 cases (64.6%), *C. bovis* in 22 (27.8%), *C. ryanae* in four (5.0%), *C. andersoni* in one (1.3%) and *C. xiaoi* in one (1.3%). In calves, 49 cases (68.1%) of *C. parvum* were detected, while *C. bovis* was identified in 19 cases (26.4%), *C. ryanae* in three (4.2%) and *C. xiaoi* in one (1.3%). In cows, *C. bovis* was observed in three cases (42.8%) *C. parvum* in two (28.6%), *C. andersoni* in one (14.3%) and *C. ryanae* also in only one case (14.3%) (Table 2). Sequence analysis of *18S* rRNA for *C. parvum* isolates, revealed 100% nucleotide identity to the AH006572 reference sequence for 51 sequences (accession numbers MW947282-MW947332). Similar sequence analysis for *C. bovis* isolates, revealed 100% nucleotide identity to AB777173 reference sequence for 20 sequences (MZ021416-MZ021425, MZ021427-MZ021429, MZ021431, MZ021433-MZ021434, MZ021436, MZ021438-MZ021440 and MZ021443) and 99% nucleotide identity to the AB777173 reference sequence for two sequences (MZ021435 and MZ021441). *C. ryanae* isolates exhibited a 100% nucleotide identity to the FJ463193 reference sequence for four sequences (MZ021430, MZ021432, MZ021437 and MZ021442). Lastly, sequence analysis for *C. andersoni* isolate, revealed 100% nucleotide identity to the AB513856 reference sequence for the one sequence (MZ021426) while the *C. xiaoi* isolate (MZ021416) exhibited a 100% nucleotide identity to the FJ896046 reference sequence (Figure 2).

#### 3.2.3. The Netherlands

Out of the 69 samples shown to be *Cryptosporidium* spp. positive in the Netherlands, the majority of those (59 cases; 85.5%) were assigned as *C. parvum* while the remaining samples were identified as *C. bovis*, (6 cases; 8.7%) and *C. ryanae* (4 cases; 5.8%). In calves, *C. parvum* was identified in 56 animals (86.2%), *C. bovis* in five (7.7%) and *C. ryanae* in four (6.1%). Out of the four positive cases in adult cows, three (75.0%) were identified as *C. parvum*, while the remaining one was *C. bovis* (25.0%) (Table 2). Further sequence analysis of the *18S* rRNA gene for the *C. parvum* isolates, revealed 100% nucleotide identity to the AH006572 reference sequence for the 59 sequences detected in this study (accession numbers MW947378-MW947436). Sequence analysis for the *C. bovis* isolates, revealed 100% nucleotide identity to the AB777173 reference sequence for six samples (MZ021464-MZ021467 and MZ021469-MZ021470). Sequence analysis of the *C. ryanae* isolates presented a 100% nucleotide identity to the reference sequence FJ463193 for four cases (MZ021462-MZ021463, MZ021468 and MZ021471) (Figure 2).

#### 3.2.4. *Cryptosporidium parvum* Subtyping through gp60 Molecular Analysis

**Figure 3:**
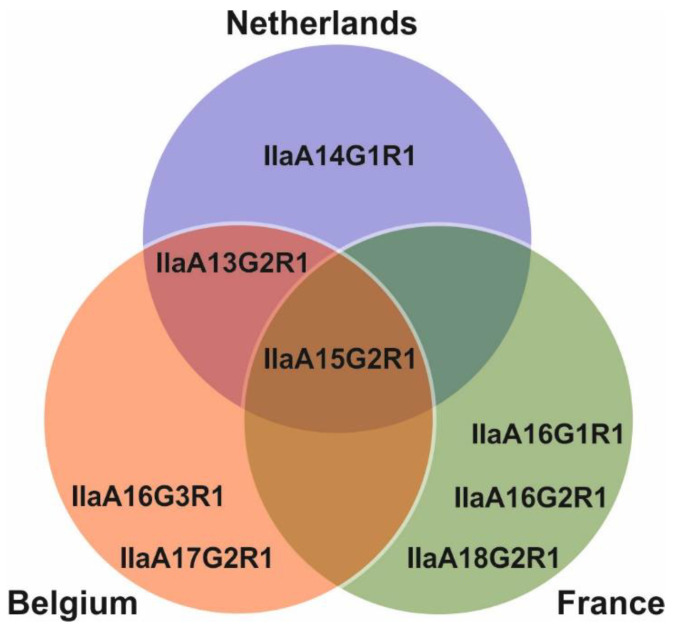
Venn diagram with all observed *C. parvum* gp60 subtypes across Belgium, France, and the Netherlands.

Using *18S* rRNA sequencing and phylogenetic analyses, a total of 155 samples were identified as *C. parvum* positive. PCR products of the *gp60* gene were successfully obtained for 137 (88.4%) of these cases. Sequence analysis and subsequent subtyping revealed the presence of eight different subtypes belonging to the IIa subtype family (Figure 3).

At least one subtype of the IIa family was found to circulate in 70% (*n* = 20) of farms in the Netherlands, 70.6% (*n* = 17) in Belgium and 70.0% (*n* = 20) in France. Regarding, the distribution of subtypes between calves and cows, in the Netherlands, all 47 (100%) of the subtypes identified were solely found in calves, while in Belgium 34 (87.2%) subtypes were described in calves and the remaining five subtypes (12.8%) in adults. Lastly, for French farms, 46 subtypes (97.9%) were identified in calves and only one subtype was identified in adults (2.1%) (Table 1 and Table 2).

Subtype IIaA15G2R1 (100% identity to the reference sequence MK099855) was present in all three countries and was also the most widespread. In the Netherlands, Belgium, and France, this subtype was identified in 89.4% (42 out 47), 66.7% (26 out of 39), and 63.8% (30 out of 47) cases, respectively. In the Netherlands, this subtype was found in 70.6% (12 out of 17) farms which tested positive for *C. parvum*, while in Belgium and France this subtype was found in 64.3% (9 out of 14) and 60.0% (9 out of 15) farms, respectively. In the Netherlands, this subtype was only reported in calves, in 89.4% (42 out 47) infections, while in Belgium 67.7% (23 out of 34) calves and three out of five (60.0%) adults were found to have this subtype. Lastly, in France, this subtype was only detected in calves, in 65.2% (30 out of 46) infections.

The second most reported subtype in this study was IIaA16G2R1 (100% identity to the reference sequence MG516787), with all 12 isolates exclusively identified in France. This subtype was observed in 20% (3 out of 15) French farms that tested positive for *C. parvum.* Its presence was mainly in calves (23.9%; 11 out of 46 infections), with only one report in an adult cow. The third most abundant subtype was IIaA13G2R1 (100% identity with reference sequence MN815775), with 11 isolates distributed through the Netherlands and Belgium. This subtype was found in just 1 out of the 17 (5.9%) Dutch farms which tested positive for *C. parvum*, and only in three calves out of 47 (6.4%). In Belgium, this subtype was also found in just one out of 12 (8.3%) farms which tested positive for *C. parvum*, with six out 34 (17.6%) calves and two out five (40.0%) cows testing positive for this subtype. The remaining five subtypes were: IIaA14G1R1, which was only observed in the Netherlands (99% identity to the reference sequence, AM937017 missing an adenine nucleotide at position 45); IIaA17G2R1 and IIaA16G3R1, which were only observed in Belgium (100% identity to the reference sequences, MG516783 and DQ192506, respectively); and IIaA16G1R1 and A18G2R1, which were only observed in France (100% identity to the reference sequences, KJ158747 and MK391451, respectively). All these subtypes were found in only a single farm and exclusively in calves (Figure 1).

## 4. Discussion

*Cryptosporidium* is the causative agent of cryptosporidiosis in humans and other animals. In the farming industry, cryptosporidiosis is a major concern among livestock. *C. parvum,* in particular, is considered to be a major cause of disease in neonatal calves, resulting in profuse diarrhoea and in extreme cases even death [13]. Herein, we employed molecular techniques based on *18S* DNA and *gp60* gene analysis aimed to assess *Cryptosporidium* spp. prevalence as well as perform subtyping of *C. parvum* among cattle in several dairy farms distributed across three countries. The contribution of several factors in *C. parvum* spreading was also evaluated. Our analyses revealed that the average prevalence of *Cryptosporidium* spp. in screened farms, was 93.0%, with France having the highest at 100%, followed by Belgium at 94.1% and the Netherlands at 85.0%. These results are in line with previous epidemiological studies recorded in beef and dairy farms in France. For instance, Follet et al., (2011) reported the presence of *Cryptosporidium* spp. in all examined beef farms following molecular detection using the *18S* rRNA gene, while Mammeri et al., (2019) described the presence of *Cryptosporidium* spp. on 92.3% of dairy farms using direct immunofluorescence assay (DFA) screening. Interestingly, in Belgium, the only published study in dairy farms reported the presence of *Cryptosporidium* spp. on 32 out of 100 farms (32.0%) using quantitative immunofluorescence assay (IFA) for parasite detection [43]. In the Netherlands, the only study carried out to assess *Cryptosporidium* spp. prevalence in cattle did not provide any information on *Cryptosporidium* spp. per farm occurrence [44]. Similar prevalence studies in Europe, employing nested PCR targeting the *18S* gene, documented less overall occurrence of *Cryptosporidium* spp. with 44.5% in Spanish dairy and beef farms, 62.3% in dairy farms of Estonia [50,51] and, in the Czech Republic, 79.2% in calves and 30.4% in cows [52,53]. In Germany and Italy, following microscopy methods for *Cryptosporidium* spp. detection, it was also observed a lower prevalence of the parasite at farm level with 68.2% in Italian dairy farms and a 70.0% prevalence in German calf farms [54,55]. A common issue between all these studies is the lack of consistent methods for sampling and parasite detection, which will allow accurate comparisons between them while avoiding detections bias.

At the individual level, by country, Belgium, France and the Netherlands totalled 18.8%, 22.6%, and 17.3% prevalence, respectively, of *Cryptosporidium* spp. infection out of all sampled animals. Past studies in France reported higher *Cryptosporidium* spp. infections in beef and dairy calves (ranging from 41.5 to 100%) [36,37,39,41,42,56]. Moreover, previous reports from Belgium also demonstrated slightly higher infections (37.0%) in dairy calves while, to our knowledge, no such information is available for the Netherlands [43]. In nearby European countries, such as Germany, United Kingdom, Austria, Spain, and Italy prevalence of *Cryptosporidium* spp. in beef and dairy calves assessed using molecular, immunological or microscopy screening, varied between 16.7% and 100% [51,54,55,57,58]. These data seem to correlate with equivalent findings worldwide where livestock parasite infections appear to lessen with the increase in age of cattle [19,27,59,60,61,62,63,64,65]. However, two recent studies carried out in dairy and beef farms in the United Kingdom pointed towards much higher infection rates in adult cattle with a reported *Cryptosporidium* spp. prevalence of roughly 80.0% in both studies, which was attributed to improved sensitivity in methods used during both investigative works [58,66]. The observed variances in reported infections might stem from distinct geographical locations in association with climate variations but also due to differences linked to the study design, with factors such as sample size, age, herd size, total number of farms investigated, farm management practices and screening methods applied playing an important factor [13].

Age-related infection predisposition to different *Cryptosporidium* species was also observed in our results after nested PCR analysis of the *18S* gene. *Cryptosporidium parvum* was clearly the dominant species in calves in all three countries, followed by *C. bovis*, *C. ryanae* and *C. xiaoi*, the latter having been observed in a single calf in France. Previous molecular studies conducted in dairy and beef calves in France and Belgium support our findings, with *C. parvum* being also the predominant infective species in pre-weaned beef and dairy calves [36,37,40,43]. Other molecular studies across Europe also seem to indicate *C. parvum* as the dominant *Cryptosporidium* species in pre-weaned calves [50,51,52,54,58,67]. The presence of *C. bovis* was less prominent in calves from this study, but this species has been reported in some parts of Europe and across the world as the major infecting species in beef and dairy calves [25,68,69,70,71,72,73,74,75,76,77]. *Cryptosporidium ryanae* was sporadically detected in calves across the three countries, which seem to be consistent with other molecular studies performed in beef and dairy farms in France and in other parts of Europe [25,36,41,50,51,52,58,74]. This species was, to our knowledge, observed for the first time in cattle from the Netherlands and Belgium. Both *C. bovis* and *C. ryanae* are mostly associated with infections in post-weaned calves and have yet been linked with cryptosporidiosis illness [13,78]. *Cryptosporidium xiaoi*, the host-specific species for sheep [79], was only recorded in one calf in France herein. Reports of infection in cattle are scarce; however, this species is reported occasionally in Jordan, China and Spain [51,80,81]. In Ireland, infections in cattle were reported as *C. bovis*/*C. xiaoi* due to the inability of distinguishing the two at the *18S* rRNA gene level [79,82]. Previous cattle infections with *C. xiaoi* were either attributed to cross-infections between livestock sharing the same grazing fields or due to contact with contaminated water [51,80]. To our knowledge, none of the farms included in this project had any association with other (small ruminants), thus the source of *C. xiaoi* remains unresolved.

The preponderance of *C. parvum* infections in calves has been debated before, with some studies suggesting that calves with *C. parvum* infections shed oocysts at a higher density when compared to other infective species, such as *C. bovis* or *C. ryanae*. The increased shed could lead to a preferential DNA amplification of the dominant *C. parvum* in a sample during *18S* rRNA gene-targeted PCR while, at the same time, masking the infection by other *Cryptosporidium* species and suppressing their detection [59,83,84,85]. Previous molecular studies in cattle mention *C. andersoni* as the primary force behind *Cryptosporidium* infections in adult cows [18,27,59,86]. However, in the present study, *C. parvum* was the dominant species infecting cows in the Netherlands (75%) and Belgium (70%), which was similar to what was reported in two recent studies carried out in United Kingdom cattle farms, which might indicate that adults shed *C. parvum* oocysts more frequently than previously thought [58,66]. Additionally, reports from Spain also documented lower *C. andersoni* infections in adult cows in cattle farms and instead found *C. bovis* as the main species present in adult cattle, as observed in our study in France [51]. These results might indicate that *C. andersoni* is not as predominant in adult cattle as previously thought, and the established *Cryptosporidium* spp. age-related prevalence is not necessarily uniform across different regions.

To further explore the genetic diversity within *C. parvum* and assess potential zoonotic transmission, subtype assessment was undertaken through *gp60* locus analysis. In total, eight different subtypes were detected across the three countries and, notably, all belonged to the IIa zoonotic allelic family. The IIa allele is exceptionally prevalent in young cattle and has been linked with various occurrences of zoonotic transmission (both sporadic and outbreak cases) of *C. parvum* in Europe [6,35,87]. Subtype IIaA15G2R1 was found to be the most prevalent in all countries with the Netherlands reporting a total prevalence rate of 89.4%, Belgium at a total of 66.7% and France a total of 63.8%. In western mainland Europe, the IIaA15G2R1 subtype was previously described as the most prevalent in cattle [36,37,40,42,43,44]. Similar observations were reported in other European countries, including Portugal, Italy, Spain, Germany, Czech Republic, Austria, Slovenia, and the United Kingdom [20,51,52,54,55,57,66,67,88] and in other parts of the world such as Japan and Uruguay [89,90]. IIaA15G2R1 is deemed as the most prevalent IIa subtype in humans in developed countries, with several cases of human cryptosporidiosis linked to it [6,44,67,87,88,91,92,93]. Several previous outbreaks with this subtype in the United Kingdom were traced back to contact between farm animals and humans pointing towards zoonotic potential [35]. In fact, it has been speculated that this subtype is hyper-transmissible, which might explain its preponderance in zoonotic infections across the world [3].

The second most documented subtype was IIaA16G2R1, with all occurrences being detected in French farms, and with a prevalence of 25.6%. This subtype was previously described in cattle of France with a much lower prevalence of just 3.9% [36]. Interestingly, this subtype was also previously observed in cattle located in Belgium and the Netherlands [43,44]. Cattle infected with this subtype was also previously found in other parts of Europe, including Portugal, Germany, Spain and Estonia [50,55,88,94]. IIaA16G2R1, has been known to cause sporadic human cryptosporidiosis in a few countries, namely New Zealand, Canada, Spain, and Jordan [91,92,95,96,97].

The third most abundant subtype in our study was IIaA13G2R1, which occurred only in the Netherlands and Belgium, with a total prevalence of 6.4% and 20.5%, respectively. This subtype has been found previously in both countries at lower frequencies than the ones reported herein, with a prevalence of 1.5% in both the Netherlands and Belgium [43,44]. From all other European countries, so far this subtype has only been reported in the United Kingdom [20]. This *C. parvum* subtype appears to be uncommon among calves with only Algeria, Turkey and Canada reporting its occurrence [98,99,100]. IIaA13G2R1 also has a low prevalence in humans with confirmed cases in Malaysia, South Korea, New Zealand, and Canada and thus its zoonotic potential is considered low [91,92,93,101].

The remaining five subtypes detected in this study had a low prevalence in all the studied countries, with less than five occurrences in each. To our knowledge, subtype IIaA14G1R1, which was only detected in the Netherlands, was for the first time described in this country and its lower prevalence (4.2%) seems consistent with other reported frequencies in cattle across various European countries such as Germany, Poland, Austria, and Estonia [50,55,57,102]. Cryptosporidiosis cases in humans for this subtype have been documented in New Zealand, Canada, Slovenia and Slovakia [67,91,92,103].

Subtypes IIaA16G3R1 and IIaA17G2R1 were only found in Belgium and were for the first time described in this country, with a 7.7% and 5.1% prevalence, respectively. Subtype IIaA16G3R1 presence was previously described in France and the Netherlands, with similar frequencies [36,40,44]. Subtype IIaA17G2R1 was only observed before in the Netherlands with also with a similar frequency to that observed in this study, though two studies conducted in French river and sea waters reported the presence of this subtype on fish [44,104,105]. Subtype IIaA16G3R1 has been found in cattle in several European countries, such as Germany, Italy, Spain, Poland, and the United Kingdom [20,51,54,55,102,106]. Subtype IIaA17G2R1 has been found in cattle in Slovakia, Poland, Spain, Germany, and the United Kingdom [20,55,94,102,106,107]. Both subtypes have been previously observed in humans in New Zealand, Canada, Denmark, and Iran [87,91,92,97,108].

Lastly, subtypes IIaA18G2R1 and IIaA16G1R1 were found only in France with a prevalence of 8.5% and 2.1%, respectively. Similar studies in France found both subtypes present in calves with one study reporting a similar prevalence of IIaA16G1R1 to the one reported in this present study [36], while another study found the IIaA18G2R1 to be the most prevalent in beef calves [41]. Subtype IIaA16G1R1 has also been observed before in the Netherlands, with a similar prevalence to the one reported in our study [44]. Subtype IIaA16G1R1 has also been reported in cattle from other European countries, including Sweden, the Czech Republic, Hungary, Poland, Romania, Slovenia, Germany and Estonia, [50,52,55,67,69,74,75,102,109,110], while subtype IIaA18G2R1 has been described before in cattle from Germany and the United Kingdom [58,66,106,111]. Human infections with these subtypes have also previously been detected in New Zealand, Canada, Sweden, Slovakia, Slovenia, Estonia, and the United Kingdom [35,67,91,92,103,112,113].

Through this study, only one subtype per farm was observed which might indicate endemicity at the farm level confirming results from previous studies [40,67,102]. This lack of genetic diversity may not be the rule though, with recent reports finding that more than one subtype per farm might be the norm [20,37,50,54,55]. The lack of genetic diversity per farm in our study might stem from the approach used, since conventional *C. parvum gp60* subtyping with Sanger sequence only targets the dominant subtype. The application of next-generation sequencing (NGS) and/or single cell genomics [114,115,116] could provide a more reliable way to unearth the real multiplicity of *C. parvum* within a herd and within the same host. In fact, two reports using NGS described up to ten individual subtypes per sample in cattle and human isolates [117,118].

Another aim of this study was to investigate the possible role of mothers in transmitting the parasite to their newborn calves. Previous investigations on the subject were conducted before the advent of molecular subtyping tools thus no conclusions could be made. A recent paper addressed this issue with *gp60* molecular subtyping [58]. The subtype analysis in our study and in agreement with the previous *18S* rRNA data analysis yielded a much higher amount of *C. parvum* genetic information in calves than in adults. After *gp60* analysis, only two pairs of adults and calves were found to share the same *C. parvum* subtype, one pair in farm 5 and the other in farm 11, both located in Belgium. This suggests that there is no clear link for maternal transmission of the parasite. A similar and recently published work also looked into this hypothesis and did not find a strong link in the spread of *C. parvum* between adults and calves, finding that calves and adults shed different *C. parvum* subtypes [58]. However, it is possible that standard *gp60* molecular and Sanger sequencing methods employed in this study might not have been discriminatory enough to uncover the true diversity of subtypes due to bias [116,117,118]. Moreover, sample collection occurred at a single time point instead of several. Thus the possibility of various additional subtypes going undetected due to differential shedding cycles cannot be excluded [58]. Although no strong evidence could be gathered regarding the source of infection of *Cryptosporidium* spp. within the farms, recent studies have started to investigate alternatives sources of transmission from outside the farms. For instance, zoonotic *C. parvum* subtypes sampled in cattle from UK farms were identical to those circulating in wildlife nearby, particularly in birds and deer [20,66]. Remarkably, the same zoonotic *C. parvum* was also found in water bodies within/nearby the farms thus water might be an alternative route of *Cryptosporidium* transmission in cattle and, eventually, transmission to humans. Thus farmers should consider implementing better water and farm management systems to prevent further environmental spread and contamination [20,66]. Moreover, although *C. parvum* transmission and infection are largely associated with mammals, recent studies conducted in aquatic environments highlighted its presence in edible fish (sea and freshwater), raising awareness for the importance of cattle as a source of environmental contamination and dispersion of *Cryptosporidium* spp. oocysts, since most of the fish were affected by the *C. parvum* subtype IIa [104,105]. Therefore, suitable farm practises that improve animal well-being and decrease occupational risks to humans in close contact with cattle should be implemented while better farm management systems to prevent further environmental spread and contamination should also be applied.

## 5. Conclusions

This study provides a detailed view of *Cryptosporidium* spp. prevalence across dairy cattle farms in Western Europe and is the first study in dairy farms in the Netherlands. Our findings indicate that *Cryptosporidium* spp. is widespread across dairy farms, with zoonotic *C. parvum* being the dominant species detected in calves across all the three countries included in this study. In addition, all the *C. parvum* subtypes identified in this study have been linked with cryptosporidiosis in humans, highlighting the potential of cattle as a reservoir for *C. parvum*. Our study also provides evidence that it is unlikely that adult cattle play a role as a source of infection, as sharing of *C. parvum* subtypes between adults and calves were only documented in two cases. However, it is important to note that other sources of infection could not be ruled out. Thus, follow-up studies should be conducted to assess cross-border and worldwide *Cryptosporidium* spp. prevalence, epidemiology, and transmission while considering a one-health approach on tackling cryptosporidiosis.

## Figures and Tables

**Table 1:** Distribution of *Cryptosporidum* spp. and *C. parvum* subtypes in all dairy cattle from the Netherlands, Belgium and France included in this study.

Country	Farm ID	Number of Screened/Positive Animals		*C. parvum*		*C. bovis*		*C. andersoni*		*C. ryanae*		*C. xiaoi*		*gp60* Subtypes of *C. parvum* (n)	
		Calf	Cow	Calf	Cow	Calf	Cow	Calf	Cow	Calf	Cow	Calf	Cow	Calf	Cow
**Belgium**	BE1	7/3	7/0	3	-	-	-	-	-	-	-	-	-	IiaA15G2R1 (3)	-
	BE2	20/5	21/3	5	1	-	1	-	-	-	1	-	-	IIaA15G2R1 (5)	IIaA15G2R1 (1)
	BE3	9/4	11/0	3	-	-	-	-	-	1	-	-	-	IIaA17G2R1 (2)	-
	BE4	9/4	10/1	4	1	-	-	-	-	-	-	-	-	IIaA15G2R1 (3)	IIaA15G2R1 (1)
	BE5	10/4	10/1	2	1	1	-	-	-	1	-	-	-	IIaA15G2R1 (2)	IIaA15G2R1 (1)
	BE6	10/1	10/1	1	1	-	-	-	-	-	-	-	-	IIaA15G2R1 (1)	-
	BE7	10/4	10/0	2	-	1	-	-	-	1	-	-	-	-	-
	BE8	10/6	10/1	2	-	4	1	-	-	-	-	-	-	IIaA15G2R1 (2)	-
	BE9	10/1	10/0	1	-	-	-	-	-	-	-	-	-	IIaA15G2R1 (1)	-
	BE10	10/1	8/0	-	-	1	-	-	-	-	-	-	-	-	-
	BE11	10/6	10/2	6	2	-	-	-	-	-	-	-	-	IIaA13G2R1 (6)	IIaA13G2R1 (2)
	BE12	7/3	1/0	-	-	3	-	-	-	-	-	-	-	-	-
	BE13	10/4	10/0	4	-	-	-	-	-	-	-	-	-	IIaA15G2R1 (4)	-
	BE14	10/3	10/0	3	-	-	-	-	-	-	-	-	-	IIaA16G3R1 (3)	-
	BE15	10/0	9/0	-	-	-	-	-	-	-	-	-	-	-	-
	BE16	10/2	10/1	-	1	2	-	-	-	-	-	-	-	-	-
	BE17	8/2	8/0	2	-	-	-	-	-	-	-	-	-	IIaA15G2R1 (2)	-
**France**	FR18	9/0	9/1	-	1	-	-	-	-	-	-	-	-	-	IIaA16G2R1 (1)
	FR19	11/3	11/0	3	-	-	-	-	-	-	-	-	-	IIaA15G2R1 (3)	-
	FR20	10/5	10/0	5	-	-	-	-	-	-	-	-	-	IIaA15G2R1 (5)	-
	FR21	10/6	10/0	6	-	-	-	-	-	-	-	-	-	IIaA16G2R1 (6)	-
	FR22	10/4	10/1	-	-	3	1	-	-	-	-	1	-	-	-
	FR23	10/7	10/1	7	1	-	-	-	-	-	-	-	-	IIaA15G2R1 (7)	-
	FR24	10/4	10/3	-	-	4	2	-	1	-	-	-	-	-	-
	FR25	10/4	10/0	3	-	1	-	-	-	-	-	-	-	IIaA15G2R1 (3)	-
	FR26	10/7	10/0	5	-	1	-	-	-	1	-	-	-	IIaA16G2R1 (5)	-
	FR27	9/5	9/0	-	-	4	-	-	-	1	-	-	-	-	-
	FR28	9/1	10/1	-	-	1	-	-	-	-	1	-	-	-	-
	FR29	5/2	5/0	2	-	-	-	-	-	-	-	-	-	IIaA15G2R1 (2)	-
	FR30	10/4	10/0	1	-	3	-	-	-	-	-	-	-	IIaA16G1R1 (1)	-
	FR31	3/3	3/0	3	-	-	-	-	-	-	-	-	-	IIaA15G2R1 (3)	-
	FR32	4/1	4/0	1	-	-	-	-	-	-	-	-	-	-	-
	FR33	7/3	7/0	3	-	-	-	-	-	-	-	-	-	IIaA15G2R1 (3)	-
	FR34	10/3	10/0	2	-	1	-	-	-	-	-	-	-	IIaA15G2R1 (2)	-
	FR35	11/4	11/0	4	-	-	-	-	-	-	-	-	-	IIaA18G2R1 (4)	-
	FR36	9/5	9/0	4	-		-	-	-	1	-	-	-	IIaA15G2R1 (2)	-
	FR37	8/1	7/0	-	-	1	-	-	-	-	-	-	-	-	-
**The Netherlands**	NL38	10/0	10/0	-	-	-	-	-	-	-	-	-	-	-	-
	NL39	10/0	10/0	-	-	-	-	-	-	-	-	-	-	-	-
	NL40	10/2	9/0	2	-	-	-	-	-	-	-	-	-	-	-
	NL41	10/2	10/1	1	1	1	-	-	-	-	-	-	-	IIaA15G2R1 (1)	-
	NL42	10/3	10/0	3	-	-	-	-	-	-	-	-	-	IIaA15G2R1 (2)	-
	NL43	10/0	10/0	-	-	-	-	-	-	-	-	-	-	-	-
	NL44	10/4	10/0	3	-	1	-	-	-	-	-	-	-	IIaA15G2R1 (3)	-
	NL45	10/3	10/0	3	-	-	-	-	-	-	-	-	-	IIaA13G2R1 (3)	-
	NL46	10/5	10/1	3	1	-	-	-	-	2	-	-	-	IIaA15G2R1 (2)	-
	NL47	10/4	10/1	4	1	-	-	-	-	-	-	-	-	IIaA15G2R1 (4)	-
	NL48	10/5	10/0	5	-	-	-	-	-	-	-	-	-	IIaA15G2R1 (5)	-
	NL49	10/5	10/0	2	-	2	-	-	-	1	-	-	-	IIaA15G2R1 (1)	-
	NL50	10/6	10/0	6	-	-	-	-	-	-	-	-	-	IIaA15G2R1 (6)	-
	NL51	10/1	10/0	-	-	1	-	-	-	-	-	-	-	-	-
	NL52	10/3	10/1	3	-	-	1	-	-	-	-	-	-	IIaA14G1R1 (2)	-
	NL53	10/2	10/0	1	-		-	-	-	1	-	-	-	-	-
	NL54	10/3	10/0	3	-	-	-	-	-	-	-	-	-	IIaA15G2R1 (3)	-
	NL55	10/5	10/0	5	-	-	-	-	-	-	-	-	-	IIaA15G2R1 (4)	-
	NL56	10/6	10/0	6	-	-	-	-	-	-	-	-	-	IIaA15G2R1 (5)	-
	NL57	10/6	10/0	6	-	-	-	-	-	-	-	-	-	IIaA15G2R1 (6)	-

**Table 2:** Prevalence and distribution of *Cryptosporidium* spp. and *C. parvum* subtypes in dairy cattle from the Netherlands, Belgium, and France.

Country	Age Group	Number of Screened/Positive Animals	Genotyping and Number of Positive Samples (%)					Genotyping of *C. parvum* at the *gp60* (*n*/%)
			*C. parvum*	*C. bovis*	*C. andersoni*	*C. ryanae*	*C. xiaoi*	
**Belgium**	**Calf**	170/53	38 (73.1)	12 (23.1)	-	3 (5.8)	-	IIaA15G2R1 (23/67.7), IIaA17G2R1 (2/5.9), IIaA13G2R1 (6/17.6), IIaA16G3R1 (3/8.8)
	**Cow**	165/10	7 (70.0)	2 (20.0)	-	1 (10.0)	-	IIaA15G2R1 (3/60.0), IIaA13G2R1 (2/40.0),
	**Overall**	335/63	45 (71.4)	14 (22.2)	-	4 (6.4)	-	IIaA15G2R1 (26/66.7), IIaA17G2R1 (2/5.1), IIaA13G2R1 (8/20.5), IIaA16G3R1 (3/7.7)
**France**	**Calf**	175/72	49 (68.1)	19 (26.4)	-	3 (4.2)	1 (1.3)	IIaA15G2R1 (30/65.2), IIaA16G2R1 (11/23.9), IIaA16G1R1 (1/2.2), IIaA18G2R1 (4/8.7)
	**Cow**	175/7	2 (28.6)	3 (42.8)	1 (14.3)	1 (14.3)	-	IIaA16G2R1 (1/100.0)
	**Overall**	350/79	51 (64.6)	22 (27.8)	1 (1.3)	4 (5.0)	1 (1.3)	IIaA15G2R1 (30/63.8), IIaA16G2R1 (12/25.6), IIaA16G1R1 (1/2.1), IIaA18G2R1 (4/8.5)
**Netherlands**	**Calf**	200/65	56 (86.2)	5 (7.7)	-	4 (6.1)	-	IIaA15G2R1 (42/89.4), IIaA13G2R1 (3/6.4), IIaA14G1R1 (2/4.2)
	**Cow**	199/4	3 (75.0)	1 (25.0)	-	-	-	-
	Overall	399/69	59 (85.5)	6 (8.7)	-	4 (5.8)	-	IIaA15G2R1 (42/89.4), IIaA13G2R1 (3/6.4), IIaA14G1R1 (2/4.2)

## Data Availability

All data have been submitted to GenBank as shown in the methods section.
